# Reduced Neutrophil Count in People of African Descent Is Due To a Regulatory Variant in the Duffy Antigen Receptor for Chemokines Gene

**DOI:** 10.1371/journal.pgen.1000360

**Published:** 2009-01-30

**Authors:** David Reich, Michael A. Nalls, W. H. Linda Kao, Ermeg L. Akylbekova, Arti Tandon, Nick Patterson, James Mullikin, Wen-Chi Hsueh, Ching-Yu Cheng, Josef Coresh, Eric Boerwinkle, Man Li, Alicja Waliszewska, Julie Neubauer, Rongling Li, Tennille S. Leak, Lynette Ekunwe, Joe C. Files, Cheryl L. Hardy, Joseph M. Zmuda, Herman A. Taylor, Elad Ziv, Tamara B. Harris, James G. Wilson

**Affiliations:** 1 Department of Genetics, Harvard Medical School, Boston, Massachusetts, United States of America; 2 Broad Institute of Harvard and MIT, Cambridge, Massachusetts, United States of America; 3 Laboratory of Neurogenetics, Intramural Research Program, National Institute on Aging, Bethesda, Maryland, United States of America; 4 Laboratory of Epidemiology, Demography and Biometry, Intramural Research Program, National Institute on Aging, Bethesda, Maryland, United States of America; 5 Department of Epidemiology, Johns Hopkins Bloomberg School of Public Health, Baltimore, Maryland, United States of America; 6 Jackson Heart Study Analysis Group, Jackson State University, Jackson, Mississippi, United States of America; 7 Comparative Genomics Unit, Genome Technology Branch, National Human Genome Research Institute, Rockville, Maryland, United States of America; 8 Division of Medical Genetics, Department of Medicine, Department of Epidemiology and Biostatistics, Institute for Human Genetics, University of California San Francisco, San Francisco, California, United States of America; 9 Inherited Disease Research Branch, National Human Genome Research Institute, Baltimore, Maryland, United States of America; 10 Human Genetics Center, University of Texas Health Science Center at Houston, Houston, Texas, United States of America; 11 Laboratory of Molecular Immunology, Center for Neurologic Disease, Brigham and Women's Hospital, Boston, Massachusetts, United States of America; 12 Department of Preventive Medicine, Center for Genomics and Bioinformatics, University of Tennessee Health Science Center, Memphis, Tennessee, United States of America; 13 Department of Epidemiology, Graduate School of Public Health, University of Pittsburgh, Pittsburgh, Pennsylvania, United States of America; 14 Department of Medicine, Division of Hematology, University of Mississippi Medical Center, Jackson, Mississippi, United States of America; 15 Jackson State University, Jackson, Mississippi, United States of America; 16 Tougaloo College, Jackson, Mississippi, United States of America; 17 University of Mississippi Medical Center, Jackson, Mississippi, United States of America; 18 Division of General Internal Medicine, Department of Medicine, University of California San Francisco, San Francisco, California, United States of America; 19 Department of Epidemiology and Biostatistics, Institute for Human Genetics, University of California San Francisco, San Francisco, California, United States of America; 20 Helen Diller Family Comprehensive Cancer Center, University of California San Francisco, San Francisco, California, United States of America; 21 V.A. Medical Center, Jackson, Mississippi, United States of America; 22 University of Mississippi Medical Center, Jackson, Mississippi, United States of America; Queensland Institute of Medical Research, Australia

## Abstract

Persistently low white blood cell count (WBC) and neutrophil count is a well-described phenomenon in persons of African ancestry, whose etiology remains unknown. We recently used admixture mapping to identify an approximately 1-megabase region on chromosome 1, where ancestry status (African or European) almost entirely accounted for the difference in WBC between African Americans and European Americans. To identify the specific genetic change responsible for this association, we analyzed genotype and phenotype data from 6,005 African Americans from the Jackson Heart Study (JHS), the Health, Aging and Body Composition (Health ABC) Study, and the Atherosclerosis Risk in Communities (ARIC) Study. We demonstrate that the causal variant must be at least 91% different in frequency between West Africans and European Americans. An excellent candidate is the Duffy Null polymorphism (SNP rs2814778 at chromosome 1q23.2), which is the only polymorphism in the region known to be so differentiated in frequency and is already known to protect against *Plasmodium vivax* malaria. We confirm that rs2814778 is predictive of WBC and neutrophil count in African Americans above beyond the previously described admixture association (*P* = 3.8×10^−5^), establishing a novel phenotype for this genetic variant.

## Author Summary

Many African Americans have white blood cell counts (WBC) that are persistently below the normal range for people of European descent, a condition called “benign ethnic neutropenia.” Because most African Americans have both African and European ancestors, selected genetic variants can be analyzed to assign probable African or European origin to each region of each such person's chromosomes. Previously, we found a region on chromosome 1 where increased local African ancestry completely accounted for differences in WBC between African and European Americans, suggesting the presence of an African-derived variant causing low WBC. Here, we show that low neutrophil count is predominantly responsible for low WBC; that a dominant, European-derived allele contributes to high neutrophil count; and that the frequency of this allele differs in Africans and Europeans by >91%. Across the chromosome 1 locus, only the well-characterized “Duffy” polymorphism was this differentiated. Neutrophil count was more strongly associated to the Duffy variant than to ancestry, suggesting that the variant itself causes benign ethnic neutropenia. The African, or “null,” form of this variant abolishes expression of the “Duffy Antigen Receptor for Chemokines” on red blood cells, perhaps altering the concentrations and distribution of chemokines that regulate neutrophil production or migration.

## Introduction

A large proportion of healthy African Americans have been observed to have a white blood cell count (WBC) that is persistently lower than the normal range defined for individuals of European ancestry [1]–[5]. This condition, called “benign ethnic neutropenia”, can have important effects on medical decision-making, since WBC is a valuable indicator of immunocompetence, infection, and inflammation. To seek the genetic basis of benign ethnic neutropenia, we recently carried out an admixture mapping analysis in which we identified a locus on chromosome 1 where local inheritance of African or European ancestry is sufficient to account entirely for the epidemiological differences in WBC levels between African Americans and European Americans [6]. By genotyping samples from two epidemiological cohorts—the Health Aging and Body Composition Study (Health ABC) and the Jackson Heart Study (JHS)—at a panel of markers that were extremely differentiated in frequency between Africans and Europeans, we identified an approximately 900 kilobase locus on chromosome 1 (99% credible interval of 155.46–156.36 Mb) where individuals with low WBC had increased African ancestry compared with the average in the genome.

In the present study, we narrowed the region of association from 900 kb to a single base pair substitution that is likely to have a strong effect on variation in WBC. To achieve this, we increased our sample size from 1,550 in the initial study to 6,005, by pooling samples from the Jackson Heart Study (JHS), the Health ABC Study, and the Atherosclerosis Risk in Communities (ARIC) Study. We found that neutrophil count is responsible for the vast majority of the WBC association at the locus, and therefore focused on neutrophil count in the current analysis. We also showed that the genetic change that is probably responsible is the Duffy Null polymorphism (rs2814778, also called *FY+/−*), which is already known to protect individuals of African descent against *Plasmodium vivax* malaria infection [7],[8], and which has recently been associated with susceptibility to HIV infection and rate of progression to AIDS [9]. Our identification of this polymorphism as the probable cause of benign ethnic neutropenia should prompt further investigation of its effects on hematopoiesis and immunity.

## Results

### Merging Samples across the JHS, ARIC, and Health ABC Studies

We pooled 6,005 African American samples from three cohort studies: the Jackson Heart Study (JHS), the Atherosclerotic Risk in Communities (ARIC) Study, and the Health, Aging and Body Composition (Health ABC) Study. For each sample, we required a high quality genome-wide admixture scan (Materials and Methods), a genotype at SNP rs2814778, body mass index (BMI), age, gender, and a full differential white blood cell count (with the exception that for Health ABC samples we did not require a measurement of bands).

**Figure 1:**
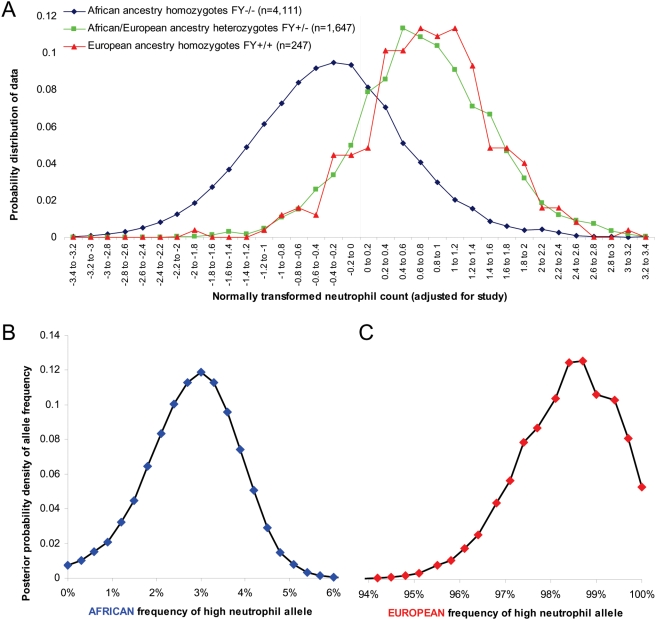
Relationship between ancestry and the distribution of neutrophil count. (A) Distribution of normally transformed absolute neutrophil count for the three classes of genotype at rs2814778. Individuals who are homozygous for the null allele have distinctly lower neutrophil count (−0.35±0.89 standard deviations compared with the mean) than individuals who are carriers for the functional allele (0.76±0.89). We were able to place constraints on the frequency of the high neutrophil count allele in (B) West Africans, and (C) European Americans by assuming that the observed distributions of neutrophil count for each ancestry class (which we marked in practice by the genotype at rs2814778) are a mixture of distributions specified by the underlying allele frequency. The results indicate a 99% probability that the frequency is <4.9% in Africans and also a 99% probability that the frequency is >95.2% in Europeans.

To explore correlations between the genetics and the phenotype, we first used the genotype at SNP rs2814778, which occurs at position 155,987,755 in Build 35 of the human genome reference sequence, within the 99% credible interval defined by our previous admixture mapping study [6]. This SNP is also known as the “*FY+*/−” or “*Duffy*” variant, and the *FY−* allele is very highly correlated to West African ancestry. For example, it is completely fixed in frequency in West African and European American samples from the International Haplotype Map [10] (although it is not completely fixed in larger sample sizes from these populations; see below). For Figure 1 and Tables 1 and 2, we used the genotype at rs2814778 as a surrogate for ancestry because the genotype can be conveniently read out as a discrete value (0, 1 or 2 copies) rather than as a continuous value, and is extraordinarily correlated to ancestry (r^2^>0.99). Later, we demonstrate that there is in fact a slightly stronger association to neutrophil count for rs2814778 than for ancestry, which is important in showing that the *FY−* allele at this polymorphism may actually be responsible for low neutrophil counts, and is not just in admixture linkage disequilibrium with the causal allele.

**Table 1:** Comparison of phenotypic characteristics for the four sets of samples used in this study.

	Number of samples	No. homozygous for null rs2814778 allele *FY*−/*FY*−	Age (range)	BMI (range)	% Female	Average percentage of European ancestry§	Total WBC	Neutrophils	Bands	Lymphocytes	Monocytes	Eosinophils	Basophils	Ratio of absolute neutrophil count in carriers of Eur. anc. to non-carriers†	Z-score for association of European ancestry carriers to neut. level§	Corr. coefficient ρ (stand. error) between Eur. anc. carrier and neut. level†
**JHS only**	1,969	1,349	52 (21–84)	32 (15–66)	60%	18.1%	5.58	3.09	.004	1.93	.387	.138	.030	1.58	29.0	.519 (.016)
**ARIC only**	2,476	1,749	54 (44–66)	30 (14–66)	60%	17.7%	5.73	2.85	.158	2.13	.347	.167	.035	1.65	30.4	.497 (.014)
* **JHS-ARIC overlap**	902	617	59 (50–74)	31 (18–59)	67%	17.8%	5.46	2.87	.080	1.94	.359	.145	.034	1.64	21.5	.568 (.021)
**Health ABC**	658	396	73 (69–80)	29 (16–43)	62%	21.3%	5.59	2.96	Not avail	1.93	.473	.172	.059	1.62	16.6	.532 (.026)
**Pooled**	6,005	3,958	56 (21–84)	30 (14–66)	61%	18.2%	5.63	2.94	.088	2.02	.376	.155	.036	1.62	49.7	.519 (.009)

Note: For the differential white blood cell counts, we present absolute values, obtained by multiplying the differential counts (expressed as a percentage of total WBC) by the total WBC. The Health ABC study did not obtain band counts.

*: For the JHS-ARIC overlap samples, the values of all phenotypes (age, BMI, and differential white blood cell counts) are averaged between the JHS and ARIC baseline measurements, taken an average of 14 years apart. Analyses in all other groups use a single measurement at the cohort baseline.

**§:** Average percentage of European ancestry was estimated using the ANCESTRYMAP software. European ancestry “carrier status” was defined by rs2814778 genotype.

**†:** Neutrophil count is used to assess the genetic association, since it accounts for essentially all the correlation to the chromosome 1 locus and thus provides the best measure of association (text and Table 2). Association analyses are carried out on the normally transformed rank-ordered values of the absolute neutrophil count. Standard errors on the correlation coefficients are obtained by a leave-1-out jackknife analysis.

**Table 2:** Effect of the chromosome 1 locus on white blood cell counts.

	Raw values of differential white blood cell counts				Rank-order transformation of differential WBC counts				Correlation (ρ) of WBC rank-order to ancestry	
					Z-scores for association tests					
	African ancestry homo-zygotes (n = 4,111) (std. err.)	Afr/Eur ancestry hetero-zygotes (n = 1,647) (std. err.)	European ancestry homo-zygotes (n = 247) (std. err.)	Ratio of 1,894 Eur ancestry carriers to 4,111 Afr ancestry homozygotes	Afr anc. homozy-gotes vs. Afr/Eur hetero-zygotes	Afr anc. homozy-gotes vs. Eur anc. homo-zygotes	Afr/Eur anc heter-ozygotes vs. Eur anc. Homo-zygotes*	Afr anc. Homo-zygotes vs. Eur ancestry carrier*	ρ between European ancestry carrier and phenotype*	ρ between Eur ancestry carrier and phenotype correcting for neut. level †
**Total WBC**	5.085 (.024)	6.798 (.045)	6.847 (.109)	1.34	39.4	19.4	0.7	41.6	.458	Not significant
**Neutrophils**	2.459 (.017)	3.982 (.034)	4.013 (.084)	1.62	47.2	23.2	0.6	49.7	.519	Not applicable
**Bands**	0.080 (.004)	0.107 (.008)	0.100 (.017)	1.32	0.6	0.1	−0.1	0.6	Not significant	Not significant
**Lymphocytes**	1.995 (.012)	2.06 (.018)	2.081 (.048)	1.03	3.9	2.0	0.3	4.2	.054	Not significant
**Monocytes**	0.351 (.003)	0.427 (.004)	0.438 (.012)	1.22	16.9	7.6	0.5	17.8	.222	.025 (P = .05)
**Eosinophils**	0.149 (.002)	0.169 (.004)	0.157 (.007)	1.13	6.1	2.0	−0.9	6.2	.080	Not significant
**Basophils**	0.034 (.001)	0.041 (.001)	0.038 (.002)	1.17	6.7	1.1	−1.7	6.5	.085	−.034 (P = .009)

*: European ancestry at the locus has an essentially dominant effect on white blood cell counts. None of the differential counts phenotypes shows a significant difference between the 1,647 *FY*−/*FY*+ heterozygotes and 247 *FY*+/*FY*+ homozygotes after correcting for 7 hypotheses tested (Bonferroni corrected P = 0.48). However, all white blood cell counts except bands show significant associations comparing the 4,111 *FY*−/*FY*− homozygotes to the 1,894 carriers of the *FY*+ allele (Z-scores between 4.2 and 49.7 standard deviations).

**†:** After controlling for neutrophil level, there is weak additional evidence for association to the chromosome 1 locus only for monocytes and basophils. For subsequent mapping analyses we only focused on neutrophil levels, as adding these other residuals did not substantially increase the strength of the association. We do not estimate correlation coefficients of the associations that are not significant.

To test for heterogeneity in the strength of the genetic association to WBC among the different sample sets that comprised our study, we divided the samples into four groups. There were 658 samples from the Health Aging and Body Composition Study (“Health ABC”), 1,969 samples from the JHS cohort only (after randomly dropping samples until there was only one from each pedigree; “JHS only”), 2,476 samples from the ARIC cohort only (“ARIC only”), and 902 samples that overlapped between JHS and ARIC (“JHS-ARIC overlap”). For the JHS-ARIC overlap samples, we averaged all phenotype measurements, taken an average of 14 years apart at the time the participant entered each study (and having a correlation coefficient of r^2^ = 0.37), to provide a more precise estimate of the phenotype than would be available from either measurement alone.

Table 1 presents the characteristics of each of the groups of samples. We found that all sets of samples showed quantitatively similar associations to the chromosome 1 locus. In particular, for neutrophil count, individuals carrying at least one European-type (“*FY*+”) allele of rs2814778 had 1.58–1.65 times higher values, depending on the study, than individuals homozygous for African ancestry (*FY−/−*), a tight enough range that we decided to pool all four groups of samples for subsequent analyses. Despite the similar correlation of neutrophil count to local ancestry across studies, we observed that the correlation coefficient to “European carrier status” was significantly higher for JHS-ARIC overlap samples, ρ = 0.57, than for the samples for which only one measurement was made: ρ = 0.52 for JHS-only (P = 0.03 for a reduction) and ρ = 0.50 for ARIC-only (P = 0.002 for a reduction) (Table 1). This is likely to reflect a more accurate assessment of basal neutrophil count when it was measured twice and averaged over different environmental conditions (the baseline measurements in JHS and ARIC studies taken an average of 14 years apart) than when it was measured only once. In support of this hypothesis, the JHS-ARIC overlap samples contributed more per sample to the statistical signal than those measured in only one cohort: 28% more per sample on average, which we calculated by dividing the LOD score they contributed by the total number of samples.

### Dominant Effect of European Ancestry on WBC Counts

Combining all samples (n = 6,005 in total) and working with normally transformed cell counts for each white blood cell lineage (Materials and Methods) we explored how counts of total WBC and each of the 6 differential counts were associated with ancestry at the locus, using the genotype at rs2814778 as a surrogate for ancestry (Table 2).

There was strong evidence that the allele at the locus that contributed to high white blood cell count had an almost purely dominant effect. As shown in Table 2, there was no significant difference in leukocyte counts between 247 African Americans with two copies of European ancestry at this locus (*FY*+/+) and 1,647 African Americans who were *FY*+/− (P>0.08 for WBC and all differential counts). By contrast, being *FY*+/+ or +/− (1,894 African Americans) vs. *FY*−/− (4,111 African Americans) was strongly associated to counts of all white blood cell types except bands (P<<10^−4^; Table 2).

The dominant effect of European ancestry on white blood cell count is also visually apparent in Figure 1, which shows the distribution of neutrophil count for individuals grouped according to genotype at rs2814778. Persons carrying at least one *FY*+ allele had a distribution of neutrophil counts that was shifted by 1.3 standard deviations above that of persons who were *FY*−/− (this was extraordinarily statistically significant: Z = 49.7). By contrast, there was no significant difference between individuals who carried either one or two *FY*+ alleles (Z = 0.6). For further analysis, we pooled individuals who were carriers of the *FY*+ allele at this locus.

### Low Neutrophil Count Is the Main Phenotype Underlying the WBC Association

The differential white blood cell count that was most significantly associated with ancestry was absolute neutrophil count (calculated as total WBC multiplied by the percentage of neutrophils). The correlation (ρ) of normally transformed absolute neutrophil count to carrier status for the *FY*+ allele was 0.519, which was higher than that of the general WBC phenotype originally mapped to the locus [6] (ρ = 0.458). In the 952 African Americans who had absolute neutrophil counts at least 1 s.d. below the mean (roughly <1,800 /mm^3^), the proportion of *FY*+ allele carriers was reduced by more than an order of magnitude compared with the genome wide average.

Neutrophil count was responsible for the vast majority of WBC association at the locus. After controlling for neutrophil count in a regression analysis, only monocyte count (ρ = 0.025, P = 0.05) and basophil count (ρ = −0.034, P = 0.009) remained nominally associated, and these associations were not significant after correcting for the 6 hypotheses tested (Table 2). The weak evidence of association to monocyte and basophil counts may reflect a real effect, or may be a false-positive due to multiple hypothesis testing. It is also possible that the result may be an experimental artifact related to the Coulter Counter technology used to measure differential WBC. In these measurements, the positions of monocytes and basophils were near those of neutrophils in the plots used for cell classification. Even a small amount of misclassification among neutrophils, monocytes, and basophils (a couple of percent) could cause their counts to be artifactually correlated, contributing to the signals we observe in the context of measurements in large sample sizes. Since neutrophil count appears to drive at least the great majority of association, we focused on this WBC phenotype for all further analysis

### Epidemiological Impact of the Chromosome 1 Locus on Neutrophil Count

To assess whether the higher neutrophil count observed in European Americans compared with African Americans can be entirely accounted for by ancestry at the chromosome 1 locus, as is the case with total WBC [6], we examined samples from the Health ABC study (1,331 European Americans and 658 African Americans). Among African Americans who could be classified with confidence as carrying at least one chromosome of European ancestry at the locus, the absolute neutrophil count did not differ from that of European Americans (P = 0.99). Thus, genetic variation at the chromosome 1 locus was sufficient to account for the entire epidemiological difference across these populations.

The predictive effect of ancestry at the chromosome 1 locus was profound. Carrier status for the European-type (*FY*+) allele at the rs2814778 variant predicted 26.95% of the variance in normally transformed neutrophil count, which was far more than the 3.37% predicted by genome-wide European ancestry proportion. After controlling for rs2814778 genotype, there was no longer any association to genome-wide European ancestry. Similarly, after controlling for rs2814778 genotype, BMI and gender only predicted 0.79% and 0.14% of variance in neutrophil count respectively, while smoking (analyzed in JHS only) only predicted 0.8% of the variance. Age was not significantly associated to neutrophil count in our data (P = 0.25). We did not analyze other phenotypes like hypertension and coronary artery disease status for their correlation to neutrophil count. Because of the relatively weak contributions of all the non-genetic predictors we analyzed, we focused subsequent analyses on genotype at the chromosome 1 locus uncorrected for covariates.

### The Causal Allele must be >91% Differentiated between Africans and Europeans

We were able to place strong constraints on the frequency of the variant affecting neutrophil count by analyzing the distributions of neutrophil count for individuals with 0, 1 and 2 copies of European ancestry at the chromosome 1 locus, which in practice we marked by the genotype at rs2814778. The analysis in Figure 1A provides strong evidence of a dominant allele of European origin contributing to high neutrophil count. We modeled the frequency of the variant that causes high neutrophil count by defining 6 parameters. The frequency of this variant in Africans was specified as P_A_ and its frequency in Europeans as P_E_. Individuals who were homozygous for the other allele were assumed to have a normal distribution of neutrophil count with mean μ_L_ and standard deviation σ_L_, and carriers of the “high neutrophil” allele were assumed to have a normal distribution of neutrophil count with mean μ_H_ and standard deviation σ_H_. Studying a grid of values of P_A_ (Figure 1B), and another grid of values of P_E_ (Figure 1C), we found the combination of the remaining variables that provided the best fit to the data, as assessed by a chi-square goodness-of-fit statistic. Given each set of 6 model parameters, we calculated a likelihood of the data for all 6,005 individuals. This resulted in a marginal likelihood surface for P_A_ (Figure 1B) and P_E_ (Figure 1C), which we used to place constraints on these parameters.

Fitting this 6-parameter model to the data, we inferred that the frequency of the allele contributing to high neutrophil counts was <4.9% in Africans and >95.2% in Europeans (Figure 1B, C), and that the difference in frequency between populations was >91.9%. Compared to 3.54 million autosomal SNPs in the November 2006 Phase2 HapMap data set [10], there were only 115 SNPs with a frequency differentiation at least this extreme, and only one in the region of admixture association: the SNP rs2814778 (at position 155.99 Mb), the same SNP we used as a marker of ancestry. This variant already has a known phenotype—susceptibility to *Plasmodium vivax* malaria—but it had not been hypothesized to be associated with low white blood cell count until it was found to lie within this locus [6]. While rs2814778 is a plausible candidate, the locus we described previously [6] spans 900 kb, and there could in principle be other variants within this span—unreported in the literature or in genome variation databases—that have a high enough frequency differentiation to explain the signal. In what follows, we present additional lines of evidence to rule out the great majority of sites other than rs2814778 as consistent with explaining the signal.

### Combining Information across Cohort Studies Halves the Admixture Peak to 450 kb

We used four strategies to increase the height of our admixture association peak and thereby to narrow the position of the allele affecting neutrophil count. First, we used the fact that with 6,005 samples in our admixture mapping analysis pooled across three studies (Table 3), we had a greater sample size than the 1,550 samples that were used initially [6]. Second, we designed an analysis (Materials and Methods) that used all of the samples instead of just the extremes of the distribution [6]. Third, we changed the phenotype from total WBC (ρ = 0.458 correlated to ancestry at the locus) to absolute neutrophil count (ρ = 0.519) to obtain a sharper statistical signal. Finally, we genotyped the JHS samples (including the JHS-ARIC overlap samples) at additional ancestry informative markers, spaced at a density of about 1 every 400 kb across the peak, to increase spatial resolution.

**Table 3:** Stratification of samples into 12 groups for admixture mapping analysis.

Standard deviations compared with population mean	Samples	Risk model used (factor change in European ancestry vs. genome average)	LOD score at rs2814778
<−1.5	400	0.04	46.6
−1.5 to −1.0	552	0.05	64.8
−1.0 to −.75	408	0.11	35.2
−.75 to −.5	493	0.19	28.6
−.5 to −.25	557	0.3	22.9
−.25 to 0	593	0.56	7.1
0 to .25	593	0.98	0.1
.25 to .5	558	1.6	4.6
.5 to .75	492	2.5	17.5
.75 to 1	408	3.5	27.8
1 to 1.5	552	5	53.8
>1.5	399	6.5	54.0
Sum across all	6,005		363.1

**Figure 2:**
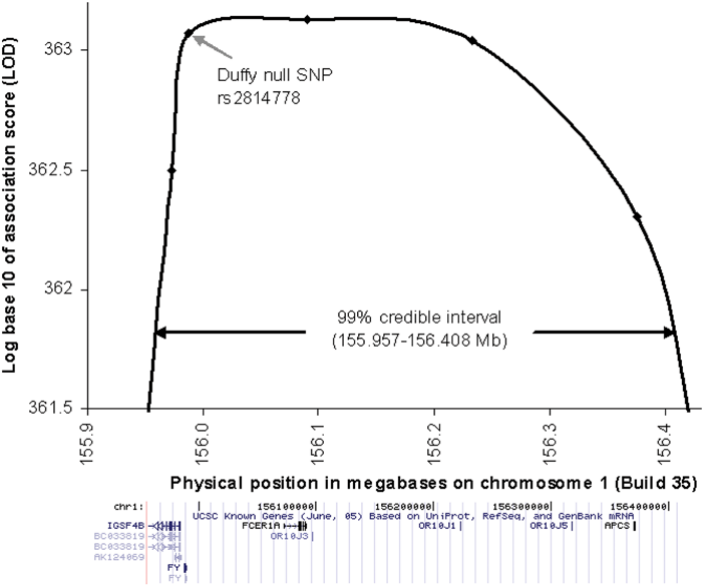
Admixture association defines a 451 kb region containing the risk allele. The LOD score for admixture association to neutrophil count shows a peak of 363.1, and a 99% confidence interval of 155.957–156.408 Mb (the region where the LOD score is within 1.44 of its maximum). The known genes under the peak are obtained using a screenshot of the “Known Genes” track from the UCSC genome browser (http://genome.ucsc.edu).

Using all 6,005 samples and the stronger phenotype of absolute neutrophil count, the LOD score (log base 10 of the Bayes score) rose to 363.1 (Table 3; Figure 2). The 99% credible interval was narrowed to ∼450 kb (155.957–156.407 kb), and still contained the Duffy null polymorphism at position 155,987,756 near the *DARC* gene (Entrez GeneID: 2532), as well as a handful of other genes listed in the lower panel of Figure 2.

### rs2814778 Is Significantly More Predictive of Neutrophil Count than Is Ancestry

We exploited the large sample size (6,005 individuals) to test whether the rs2814778 variant predicted low neutrophil count more than would be expected from the association to ancestry [6]. This is a difficult problem since the genotype at this SNP is highly correlated to ancestry. By using the ANCESTRYMAP software and the data from all 6,005 African Americans, we estimated that the frequency of FY+ allele at rs2814778 is 0.2±0.1% in Africans and 99.3±0.4% in Europeans (this frequency distribution is consistent with the allele frequencies inferred for the causal allele based on modeling of neutrophil counts in Figures 1B and 1C). Thus, if rs2814778 is the causal variant, there should be a small handful of individuals for whom the genotype at rs2814778 is discrepant with ancestry, who will be informative for our analyses.

To estimate the number of individuals who we expect to be informative for testing association of rs2814778 above and beyond ancestry, we used the fact that the cohort has 18.2% European ancestry on average (Table 2). Thus, we expected there to be about 13 individuals who are homozygous for the Duffy null allele at rs2814778 but heterozygous for European ancestry: 13 = (6005)×(2×18.2%×81.8%)×(0.7%). Similarly, we expected there to be about 8 individuals who are heterozygous at rs2814778 but homozygous for local African ancestry: 8 = (6005)×(81.8%×81.8%)×(0.2%).

To test for association to rs2814778 above and beyond ancestry, we first obtained estimates of European ancestry at the position of the SNP using the ANCESTRYMAP software [11]. We included rs2814778 in the ancestry estimation so that we could explicitly test whether the genotype at this SNP alone was more predictive of neutrophil count than this SNP plus flanking markers. This would be evidence that it was more associated than African ancestry itself. Our power to detect a signal was highest for JHS samples, which were genotyped at a high density at the chromosome 1 locus. Consistent with this observation, the 7 samples for which we could state with >50% confidence that the local ancestry was discrepant with the expectation from the rs2814778 genotype were all from JHS.

We performed three regression analyses (Table 4) to explore whether rs2814778 or ancestry status at the chromosome 1 locus was a better predictor of neutrophil count. (a) First, we obtained a χ^2^ statistic for association of carrier status for the rs2814778 *FY+* allele to neutrophil count; (b) second, we obtained a χ^2^ statistic for association of carrier status for European ancestry to neutrophil count (using the rs2814778 genotype in the estimate); and (c) third, we obtained a χ^2^ statistic for association of both predictors together. We found that there was a significant difference between the strength of association of ancestry alone and ancestry and genotype together: (c)-(b) = 15.7 (P = 3.8×10^−5^). Testing for the reverse effect of ancestry above and beyond the genotype of rs2814778 produced no signal: (c)-(a) = 0.4 (P = 0.74). These results confirm that rs2814778 is predictive of neutrophil count, above and beyond the effect of ancestry.

**Table 4:** Reduced neutrophil count is more associated to the Duffy null polymorphism than to ancestry.

	χ^2^ value for association of neutrophil count to a predictor (n = 5,997 individuals passing analysis quality filters)
**Carrier status for SNP allele** (χ^2^ from 1-variable regression)	1883.2
**Carrier status for European ancestry** * (χ^2^ from 1-variable regression)	1867.9
**SNP and ancestry** * **together** (χ^2^ from 2-variable regression)	1883.6
**Association to SNP beyond ancestry** (χ^2^ subtracting lines 3–2, one-sided test)	15.7 (P = 3.8×10^−5^)
**Association to ancestry beyond SNP** (χ^2^ subtracting lines 3–1, one-sided test)	0.4 (P = 0.74)

*: For the association tests, we used ancestry estimates that included the genotype of the SNP. This ensures that any association at the SNP is above and beyond that expected from ancestry alone.

### A Fine-Mapping Scan Fails to Find Additional Signals of Association

To search for additional alleles in the admixture peak that might be associated to neutrophil count beyond the main effect, we genotyped a dense panel of 193 SNPs across the region in 148 individuals with low neutrophil count (<−0.7 standard deviations below the mean) and 74 individuals with high neutrophil count (1.3–2.8 standard deviations above the mean). We chose only individuals for whom we were >99% confident of all African ancestry at the locus, based on genotyping information at flanking markers excluding rs2814778, so that ancestry would not be a confounder of the analysis.

**Figure 3:**
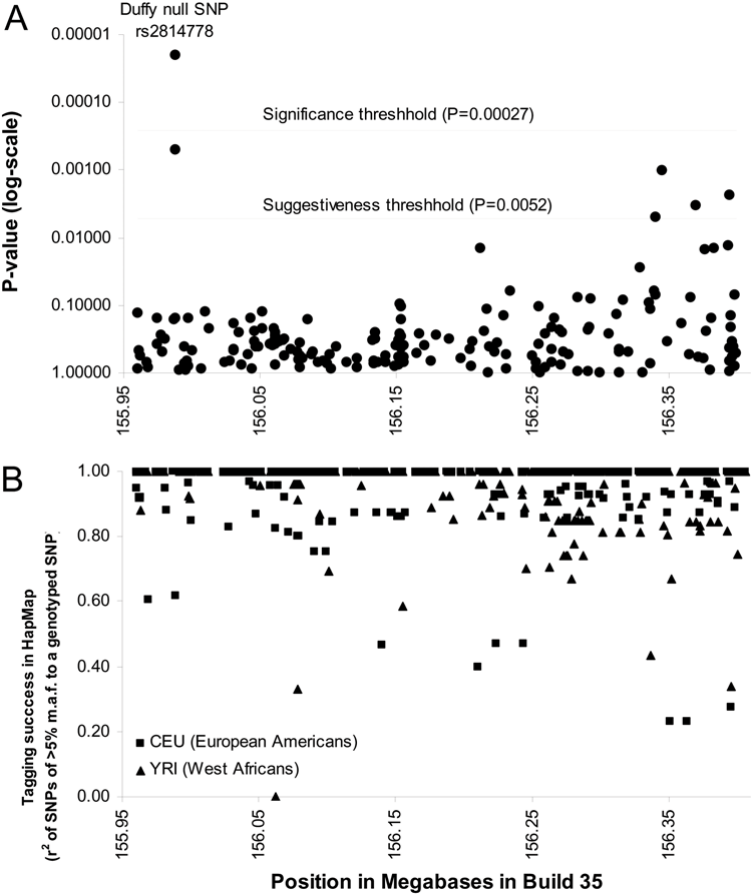
Fine mapping reveals rs2814778 as the only significant association. (A) Results of case-control association analysis for 193 SNPs genotyped in 148 individuals with low neutrophil count (<2,100/mm^3^), which we compared with 74 controls with high neutrophil count (5,000–9,000/mm^3^). All samples were selected to have a confident estimate of all African ancestry at the chromosome 1 locus (>99% probability) based on ANCESTRYMAP analysis at flanking markers outside the admixture peak. (B) HapMap SNPs of >5% minor allele frequency are well captured by this genotyping. We find that 94% of West African SNPs and 96% of European American SNPs are correlated with r^2^>0.8 to one of the SNPs we genotyped.

We genotyped these individuals for a set of SNPs chosen using Tagger [12] to capture the great majority of common variation across the admixture peak in both West Africans and Europeans (Materials and Methods). After the genotyping was complete, we had captured 94% of SNPs of >5% minor allele frequency in West Africans, and 96% of SNPs of >5% minor allele frequency in European Americans, both at a correlation of r^2^>0.8 (Figure 3B).

Case-control association analysis of these 193 SNPs identified only one, rs2814778, that was significantly associated (nominal P = 2.1×10^−5^; Figure 3A) after a Bonferroni correction for 193 multiple hypothesis tests. Thus, there was no evidence of any allele in the region that is associated to neutrophil count beyond the effect that is already captured by rs2814778.

### Genotyping of rs2814778 in >10,000 European Americans with a Neutrophil Count

We genotyped 10,062 self-identified European Americans in the ARIC study for rs2814778, searching for a decreased neutrophil count in association with the null allele. This analysis should have little power if the European American population is in Hardy-Weinberg equilibrium, since *FY*−/− homozygotes are expected to occur very rarely among Europeans: less than 1/10,000 based on the observed frequency of the null allele in this population (0.34 = 10,062×0.58%×0.58%). Interestingly, we observed 7 European Americans with *FY*−/− genotypes, a significant excess compared with expectation (P<4×10^−9^) suggesting that European Americans harbor population substructure with variable levels of African ancestry. Among the *FY*−/− homozygotes we found a non-significant reduction in WBC associated with the null allele: WBC was observed to be 5.9±2.6 for the 7 *FY*−/− homozygotes, 5.9±1.8 for the 103 *FY*+/− heterozygotes, and 6.3±1.9 for the 9,952 *FY*+/+ homozygotes (P = 0.06 with an additive model and P = 0.35 with a dominant model using 1-sided tests). Genotyping of rs2814778 in 1,339 self-identified European Americans from the Health ABC study identified 26 heterozygous individuals, and none homozygous for *FY*−/−.

### Exclusion of Most Single Nucleotide Changes in the Admixture Peak Other than rs2814778

These analyses strongly increase the likelihood that a single nucleotide change at the site of the rs2814778 polymorphism is responsible for low neutrophil counts, and provide no evidence of any other allele contributing a signal. However, these findings do not rule out the existence of undiscovered variants in the admixture peak that are differentiated enough to explain the signal. If such variants existed, then rs2814778 could simply be a marker in linkage disequilibrium with the causative variant rather than being causal itself.

We carried out an analysis in which we systematically ruled out the majority of other nucleotides in the region as potentially contributing to the signal. We examined genomic databases to identify DNA sequence fragments that are known to be of either African or European ancestry and that overlap the admixture peak, and considered nucleotides where all African chromosomes had one allele and all European chromosomes had the other. Based on our modeling in Figure 1, it is likely that the causative variant is sufficiently differentiated that it would be found by this discovery strategy.

**Figure 4:**
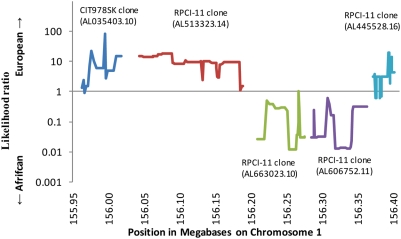
Ancestry analysis of BAC clones that are the source of the human genome reference sequence across the chromosome 1 locus. The human genome reference sequence across the admixture peak is pieced together from 5 BAC clones, which turn out to be a mosaic of European and African ancestry. To determine ancestry, we examined the haplotype of the human genome reference sequence for 284 SNPs for which data are available from the International Haplotype Map Project, and then output the ratio of the number of perfect matches to the reference sequence haplotype in 120 European American to the number of perfect matches in 120 West African chromosomes (conservatively adding 1 to the counts). Values above 10 indicate strong (>10∶1) evidence for a European haplotype, and values below 0.1 indicate strong (<1∶10) evidence for an African haplotype. RPCI-11, the individual who is the source of ∼74% of the human genome reference sequence, has two clones of entirely African and two clones of entirely European ancestry at this locus, strongly indicating that the human genome reference sequence is primarily that of an African American. We included these BAC clones in our search for polymorphisms across the admixture peak that were consistent with being fixed in frequency between European and African populations.

We mined shotgun sequencing data from 6 individuals of West African ancestry and 5 individuals of European ancestry (Materials and Methods). We restricted analysis to nucleotides for which we had a high sequence quality score (Neighborhood Quality Score of ≥40) [13], randomly sampling one sequence to represent each individual. We supplemented the shotgun data with the human genome reference sequence, which is comprised of a mosaic of sequence from 5 BAC clones across the region. We determined that 3 of the clones (one from individual CIT978SK and two from individual RPCI-11) were of European ancestry, and 2 were of African ancestry (both from RPCI-11) (Figure 4). Interestingly, since RPCI-11 was heterozygous for African and European ancestry at this locus, this individual is probably African American. Because RPCI-11 is the source for most (∼74%) of the human genome reference sequence [14], we conclude that much of the public human genome reference sequence is that of an African American, and includes a substantial amount of sequence of African ancestral origin.

We found that 82.3% of the admixture peak was covered in at least one chromosome from each population (an average of 2.2× European and 2.1× African coverage). Of the 817 SNPs we identified, 594 could be ruled out as not completely differentiated in frequency between the European and African chromosomes used in SNP discovery, an additional 79 could be ruled out as not sufficiently differentiated across populations based on data from the International Haplotype Map database [10], and 49 could be ruled out by their allele frequencies in our own follow-up genotyping of HapMap samples. Thus, 88.5% ( = (594+79+49)/817) of SNPs discovered in the admixture peak could be ruled out. This allowed us to infer that 72.8% ( = 82.3%×88.5%) of nucleotides can be excluded as causal for the observed major effect on variation in neutrophil count.

These results provide yet another line of evidence that the rs2814778 single nucleotide change may itself be causing low neutrophil levels. There are now five reasons why we believe rs2814778 is likely to be the direct cause for low neutrophil count: (1) rs2814478 falls within the ∼450 kilobase admixture peak. (2) rs2814778 contributes a signal of association above and beyond the admixture signal, showing that the true underlying causal variant is in an even narrower region around rs2814478. (3) rs2814778 is already known to have functional consequences, based for example on past molecular work showing that it affects expression of an antigen on red blood cells and thus modulates resistance to *P. vivax* malaria. (4) rs2814778 is known to have a frequency differentiation across populations that makes it consistent with the underlying causal variant; a degree of differentiation that is extremely unusual, with only 0.003% of known SNPs in the genome having a differentiation this extreme. (5) Genome sequencing data directly rule out about three quarters of other nucleotides in the admixture peak as containing the variant. (We have not carried out a similar analysis of insertion/deletion polymorphisms, which are known to occur at about a tenth the rate of SNPs.)

We caution that an association study is always correlational, and can never by itself prove a functional effect of an allele. To prove causality, it is essential to follow up any association study with biological work. Nevertheless, the present study provides the best example of which we are aware of taking association analysis to its limit, and using association analysis to demonstrate a likely causal effect. Our study justifies further work to understand the biological mechanism by which a single nucleotide change at rs2814778 probably causes reduced neutrophil counts.

## Discussion

We have used admixture mapping to localize a variant affecting neutrophil levels to a region of about 450 kb centered on the Duffy null locus. We have further shown that the underlying variant must be >91% different in frequency between West Africans and European Americans, placing it among the top 115 HapMap SNPs in the genome in terms of allele frequency differentiation (top 0.003%). Since only one SNP in the admixture peak, the Duffy null polymorphism rs2814778, is known to be this differentiated in frequency across populations, we tested this SNP for evidence of association above and beyond the ancestry effect, and found a signal (P = 3.8×10^−5^). Finally, we ruled out the great majority of nucleotides across the region, apart from rs2814778, as sufficient to cause the signal.

Methodologically, these results provide a case-example of fine-mapping in a difficult context. We have moved from an initial association signal discovered via mapping by admixture linkage disequilibrium, to a more fine-grained association based on linkage disequilibrium inherited from the ancestral African and European populations, and finally to an analysis where we systematically excluded the great majority of nucleotides in the region as contributing to the association.

The study is also novel in demonstrating the value of mapping in multi-ethnic and admixed populations. The variant could not have been mapped in non-African Americans (either Africans or Europeans), since it is nearly fixed in both populations, showing how studying diverse populations is important in biology. For example, when we genotyped rs2814778 in more than 10,000 European Americans from the ARIC study, we could not obtain a replication despite the large sample size.

The mechanism of low neutrophil count in persons homozygous for the *FY*− allele is unknown. Interestingly, Yemenite Jews also have a high frequency of the *FY*− allele [15], which we hypothesize explains the occurrence of reduced neutrophil count in this group. The term “ethnic neutropenia” has been applied to persistently low neutrophil count in both Yemenite Jews and in populations of African ancestry, and the condition is clinically similar in these two populations. Persons with ethnic neutropenia have a reduced capacity to mobilize bone marrow neutrophil reserves in response to corticosteroids, despite normal cellularity and maturation of all cell lines in bone marrow aspirates [16]–[18]. Exercise-induced increments in neutrophil counts (demargination) are also smaller in persons with “ethnic neutropenia” than in healthy volunteers. Thus, the low neutrophil count is not the result of increased sequestration of neutrophils in the marginated granulocyte pools (cells adherent to the endothelium of post-capillary venules) [19].

The *FY−* allele of rs2814778 has a −46 T to C substitution (non-coding strand) in the *Duffy Antigen Receptor for Chemokines* (*DARC*) gene, which disrupts a binding site for the GATA1 erythroid transcription factor [20]. This substitution abolishes gene expression in erythrocytes but not other cell types, such as endothelial cells of the post-capillary venules [21]. The *DARC* gene product is a seven-transmembrane receptor that selectively binds “inflammatory” chemokines of both the CXC and CC families, including, for example, *CXCL8* (interleukin 8) and *CCL5* (RANTES), both of which are involved in neutrophil recruitment [22]–[25]. Unlike related chemokine receptors with signaling function, DARC lacks a G-protein binding motif. It is nevertheless capable of internalizing bound chemokine [21], and it has been hypothesized to affect leukocyte recruitment to sites of inflammation through its role in trancytosis of chemokines through endothelial cells [24]–[26]. DARC on red blood cells might affect the number of circulating neutrophils through any of several mechanisms, for example, by modulating the concentrations of chemokines in vascular beds in the bone marrow [25], or by acting as a chemokine “sink” to limit the stimulation and extravasation of circulating neutrophils, or through other mechanisms that are not yet understood. Interestingly, *DARC*-knockout mice, which lack DARC expression not only on red blood cells but also in other tissues, do not differ from wild-type mice in peripheral blood leukocyte levels [27], but have a different phenotype, of increased bone mineral density [28]. When we tested whether an association with bone mineral density existed in African Americans in the Health ABC cohort, however, rs2814778 genotype was not significantly associated with either total (n = 1,141, P = 0.57) or femoral neck (n = 1,141, P = 0.43) bone mineral density.

The expression of DARC on erythrocytes is known to modulate chemokine levels after endotoxin treatment [29]; thus the *FY−* allele could potentially have important effects in critically ill patients with sepsis. More subtle effects on innate immunity and inflammation could also exist through DARC modulation of chemokine concentrations in specific vascular and tissue microenviroments[25]. However, we were not able to directly demonstrate an effect of the *FY−* allele on health. When we carried out tests of association of *FY−* to a wide range of phenotypes in the Health ABC Study (M. Nalls and T. Harris unpublished data), we did not find association to any phenotype. It is not surprising that health impacts of this variant are subtle, since the allele has risen to nearly 100% frequency in some African populations without being retarded in its rise by natural selection. This forms a sharp contrast with the *HBB* allele, which confers resistance to *Plasmodium falciparum* in heterozygous individuals (analogous to *FY−* conferring resistance to *Plasmodium vivax*) but causes sickle cell disease in homozygous form, and as a result has never risen to more than about twenty percent frequency in any population.

Another recent study found that the *FY−* variant of *DARC* is associated to altered rates of HIV infection and disease progression, potentially suggesting a health effect of the low neutrophil count [9]. The authors found that there is a significantly slower rate of disease progression in *FY−/−* individuals infected by HIV-1 than in carriers of the *FY+* allele. To explore this result, they performed functional studies showing that *in vitro*, HIV-1 attaches to erythrocytes via DARC and uses it as a means of transfer to target cells. They argued that such transfer might be impaired in *FY−/−* individuals, leading to slower disease progression. However, these authors also identified a second phenotype that is associated to the *FY−/−* genotype—a 40% higher rate of acquiring HIV-1 infection—that is difficult to attribute to the same mechanism, since the failure to express DARC on red blood cells might be expected to decrease access by HIV-1 to CD4-positive target cells. Potential explanations are that either low neutrophil counts or altered chemokine concentrations due the *FY−/−* genotype may have some role in modulating infection. These possibilities should be testable in the laboratory. It is also possible that differing frequencies of the *FY−* allele reflect stratification within the study population, and that differences in DARC expression are not actually involved in modulating susceptibility to infection or disease progression.

An immediate consequence of our finding is that genotyping of rs2814778 (or measuring Duffy antigen expression on red blood cells) might be used as a diagnostic guide in clinical situations in African Americans, helping to set a baseline expected neutrophil count for patients, and to guide treatment. Reduced neutrophil count has been cited as a potential cause of treatment delay and of less intensive therapy for early-stage breast cancer in African American women, perhaps contributing to ethnic disparities in breast cancer survival [30]. It also may alter the course of cytotoxic therapy for inflammatory diseases such as rheumatoid arthritis and systemic lupus erythematosus, perhaps needlessly. Finally, “ethnic neutropenia” may contribute to a diminished leukocytic response to infection [31]–[34], perhaps resulting in a lowered index of suspicion and delayed diagnosis of infection in selected patients. New studies are needed in each of these clinical settings to incorporate genotyping information from rs2814778 to help in the interpretation of neutrophil counts.

## Materials and Methods

### Human Subjects

The 6,005 human subjects for this study were drawn from three observational cohorts in which large numbers of phenotypic measurements had been made: the Jackson Heart Study (JHS) [35], the Health, Aging and Body Composition (Health ABC) Study [36], and the Atherosclerosis Risk in Communities (ARIC) Study [37]. From each of these studies, we only included African Americans for whom a complete differential white blood cell count was available, including measurement of neutrophils, bands, lymphocytes, monocytes, eosinophils and basophils. An exception was the Health ABC study, which did not provide a measurement of bands. In addition, we restricted our analysis to individuals for whom we had an admixture scan that passed all our quality control filters; for whom we had a genotype at the Duffy polymorphism, rs2814778; and for whom we had information on gender, age, and body mass index (BMI). All WBC and phenotypic measures were from baseline data collected at the time of enrollment in each study.

The JHS cohort [35] consists of 5,302 self-identified African American men and women recruited between September 2000 and March 2004 from the three counties surrounding Jackson, MS. Unrelated persons aged 35–84 were enrolled from three sources: previous ARIC participants (31% of the total), random selectees from a commercial listing (17%), and members of an age- and sex-constrained volunteer sample (30%). The remaining participants, at least 21 years old, were members of a nested family cohort. A total of 3,945 JHS participants had the required phenotypic data and were successfully genotyped for a panel of admixture mapping markers. A subset of 2,871 was included in our analysis after randomly dropping samples of related individuals until there was only one individual included per family. Of these, 1,969 were “JHS unique” samples that were present only in JHS, and 902 were “JHS-ARIC overlap” samples, representing persons who had participated in both the JHS and ARIC studies. For the “JHS-ARIC overlap” samples, we averaged the baseline measurements for each individual at the time of their entry into each cohort, an average of 14 years apart.

The Health ABC cohort [36] consists of 3,075 men and women aged 70–79 who were enrolled between April 1997 and June 1998. All were Medicare beneficiaries living near Pittsburgh or Memphis and all reported having no difficulty performing basic physical activities. Of the 1,281 participants who identified themselves as African American, 658 had complete genotype and phenotype data and were included in the current study. Of the participants who identified themselves as European American, 1,331 were analyzed for the purpose of comparison with African Americans.

The ARIC cohort [37] consists of 15,792 randomly-selected participants aged 45–64 who were recruited between November 1986 and December 1989, in roughly equal numbers, from field centers in Jackson, MS, Minneapolis, MN, Forsyth County, NC, and Washington County, MD. The cohorts of the latter three field centers represent the ethnic mix of their communities. The Jackson-based cohort (n = 3,728) was limited to self-identified African Americans, and comprised 87.4% of all African Americans in ARIC (1,626 Jackson-based participants were later enrolled in JHS). A total of 3,378 African American participants were included in the present analysis after applying all data filters. Of these, there were 2,476 “ARIC unique” participants who were present only in ARIC. There were 902 “JHS-ARIC overlap” samples as described above. A total of 10,062 European American ARIC participants were also genotyped and analyzed with respect to a single variant, rs2814778.

### Differential WBC

For all three studies, cells in EDTA-anticoagulated venous blood were counted using a Beckman-Coulter Counter (Beckman Coulter, Inc., Fullerton, CA), which combines measures of electrical conductivity and light scatter to distinguish cell lineages in suspensions of unstained leukocytes, yielding an overall WBC and relative proportions for each of six leukocyte subgroups: neutrophils, monocytes, lymphocytes, basophils, eosinophils, and “band forms”, expressed as a percentage of total WBC. Absolute counts were obtained by multiplying the differential count (a percentage) by total WBC.

To create a phenotype for analysis, all counts were rank-ordered within one of the four groups of samples (Health ABC, JHS only, ARIC only and JHS-ARIC overlap), and then assigned a percentile. An inverse normal transformation was used to translate this percentile into a normally distributed phenotype.

### Genotyping

Genotyping of African American samples on the admixture mapping panels was performed using the Illumina BeadLab platform [38], which can analyze a custom panel of 1,536 SNPs. We have developed three consecutive versions of a custom admixture mapping panel, each providing incrementally better coverage of the genome than the previous version, and all yielding excellent coverage. A total of 1,119 samples were genotyped in the “Phase 2” panel [39] (Health ABC samples and 16% of JHS samples) and 4,886 samples were genotyped in the “Phase 3” panel [6] (ARIC samples and 84% of the JHS samples). All panels include the rs2814778 polymorphism in the *DARC* gene. The genotyping of the Health ABC and JHS samples was carried out at the Broad Institute of Harvard and MIT in Cambridge as previously described [6]. Genotyping of the ARIC samples was carried out at the Center for Inherited Disease Research (CIDR) in Baltimore, MD.

Genotyping of the rs2814778 polymorphism in 10,062 ARIC European American samples and 1,339 Health ABC European American samples was done using the ABI TaqMan technology [40].

### Data Quality Checks for Ancestry Informative Markers

We used built-in data quality checks in the ANCESTRYMAP software [11],[39],[41],[42] to remove SNPs that were not appropriately intermediate in frequency in African Americans compared to the West African or European American ancestral populations, or that had evidence of being in linkage disequilibrium (LD) with each other in these ancestral populations [11]. After this filtering, the JHS samples had 1,265–1,532 SNPs available for analysis, the Health ABC study samples had 1,128–1,385 SNPs, and the ARIC study samples had 1,277–1,529 SNPs.

### Genotyping in JHS to Improve Spatial Resolution of the Chromosome 1 Peak

To refine the peak of admixture association, we used the Sequenom iPLEX platform [43] to genotype all JHS samples more densely in the region of highest interest on chromosome 1 (153.5–157 Mb in Build 35 of the reference sequence). After filtering to remove SNPs in LD in the ancestral populations or with poor genotyping performance, we had data from 9 markers across this region in JHS (rs2768744, rs2309879, rs7528684, rs1587043, rs857859, rs2814778, rs11265198, rs2494493 and rs11265352), compared with 2 in the other studies (rs2768744 and rs2814778).

### Use of rs2814778 as a Surrogate for Ancestry at the 1q23.2 Locus

The “C” allele of rs2814778 (also “*FY−*”) is known to be almost completely correlated to West African ancestry at the chromosome 1q23.2 locus. We therefore used this allele as a marker to study the epidemiological association of African ancestry to various WBC phenotypes (Figure 1 and Tables 1 and 2). In addition to using *FY−* as a surrogate for ancestry, in the final analyses of this study (Figure 3 and Table 4), we took advantage of the fact that the correlation between this allele and African ancestry, though >99%, is not perfect. Thus, we could test whether the allele is more predictive of neutrophil count than is African ancestry itself.

### Testing Other Leukocyte Counts after Controlling for the Effect of Neutrophil Count

To test for association of the chromosome 1 locus to counts of leukocytes other than neutrophils, we carried out a regression analysis between absolute counts of neutrophils and absolute counts of each of the other white blood cell types. This generated a residual value for each cell type after correcting for the effect of neutrophil count. We then carried out 2-sided tests for association of each of these residuals to carrier status for European ancestry at the chromosome 1 locus (defined as having at least one *FY+* allele at rs2814778), using Z-scores to indicate the difference, in standard deviations, in the population means between groups of samples. These Z scores can be approximately translated into a P-value by referring to the corresponding percentile in the cumulative normal distribution function.

### Narrowing the Chromosome 1 Peak by Admixture Mapping

The ANCESTRYMAP software [11] was used to better define the region of chromosome 1 associated to neutrophil count. Since the software is optimized for dichotomous traits, we divided the 6,005 samples into 12 strata (with 399–593 samples each) based on their normally transformed neutrophil counts (Table 3). For each stratum, we identified a risk model (increased probability of observing a sample with 1 or 2 copies of European ancestry compared with the expectation from the genome-wide average) that optimized the peak LOD score at the locus. This ranged from a 25-fold decrease in the relative probability of European ancestry for individuals with neutrophil counts <−1.5 standard deviations below the mean, to a 6.5-fold increase for those with neutrophil counts >1.5 standard deviations above the mean. For each stratum, the risk model and LOD score at the chromosome 1 locus are given in Table 3.

To use these strata to define an admixture peak, we carried out an admixture scan for each group separately, and then summed the LOD scores at loci interpolated every tenth of a centimorgan. The peak LOD score was 363.1 as shown in Table 3. The 99% credible interval of 155.957–156.408 Mb in Build 35 of the human genome reference sequence was determined by the region where the score was within log_10_(e^6.63/2^) = 1.44 of its maximum (Figure 2). This is calculated from a likelihood ratio test, using the fact that a χ^2^ statistic with 1 degree of freedom of 6.63 corresponds to P = 0.01.

### Testing for an Association of rs2814778 above and beyond the Admixture Association

To assess whether genotype at rs2814778 is more predictive of neutrophil count than ancestry, we calculated two numbers for each DNA sample. First, we recorded whether the individual was a carrier of the *FY+* “functional” allele ( = 1) at rs2814778, or was homozygous for the *FY−* “null” allele ( = 0) (homozygosity for the “null” allele abolishes expression of the Duffy antigen on red blood cells but not on other cell types). Second, we used the ANCESTRYMAP software [11] to estimate the probability that in the region spanning this SNP, at least one of the individual's chromosomes was of European ancestral origin. Importantly, we included the genotype of rs2814778 in the ancestry estimation. Thus, if neutrophil count were better correlated to rs2814778 genotype than to an ancestry estimate that included information from both rs2814778 and closely neighboring SNPs, it would indicate that the neighboring SNPs did not add relevant information, and that rs2814778 is either the causal variant or is in strong LD with the causal variant.

To determine whether genotype at rs2814778 or ancestry at the chromosome 1 locus was the better predictor of neutrophil status, we carried out three regressions:

- We regressed the genotype of the Duffy functional allele at rs2814778 to neutrophil count under a dominant model and calculated a χ^2^ statistic.
- We regressed inferred European ancestry at the chromosome 1 locus to neutrophil count using a dominant model and calculated a χ^2^ statistic.
- We regressed both predictors together to neutrophil count and calculated a χ^2^ statistic.

To test for evidence of an association of the genotype at rs2814778 above and beyond ancestry, we subtracted the χ^2^ statistics of (c)-(b). To test for evidence of association to ancestry above and beyond SNP genotype, we subtracted the χ^2^ statistics of (c)-(a).

### Case-Control Fine-Mapping Over the Admixture Peak in JHS Samples

To test whether there were SNPs apart from rs2814778 that contributed evidence for association at the chromosome 1 locus, we densely genotyped a subset of especially informative samples. To select cases and controls for fine-mapping, we identified individuals from JHS for whom we were >99% confident of African ancestry on both chromosomes at the admixture peak. By limiting cases and controls to persons with more confident estimates of entirely African local ancestry, it was easier to detect whether any signal of association was significant above and beyond the admixture association. For this analysis, the estimate of ancestry at the admixture peak using ANCESTRYMAP [11] excluded SNPs within the peak.

Among individuals who had >99% confidence of entirely African ancestry at the locus, we identified 696 individuals who had a “low” neutrophil count, defined based on visual inspection of the distribution as an absolute count of <2,100/mm^3^ (and corresponding to <0.7 s.d. below the population mean for the entire JHS sample). We also identified 77 individuals who had a “high” neutrophil count, defined as an absolute count of 5,100/mm^3^–9,100/mm^3^ (1.3–2.8 s.d. above the population mean). For genotyping, we selected a random subset of individuals with “low” neutrophil count, and all of the individuals with “high” neutrophil count. We successfully genotyped 148 subjects with low and 74 subjects with high neutrophil count that we could use in this analysis.

To identify additional SNPs across the admixture peak that might be associated with neutrophil levels, we used the Tagger software [12] to choose a panel of SNPs from the International Haplotype Map database [10] that captured all SNPs of >5% minor allele frequency in West African samples with a correlation of r^2^>0.8. Forcing these SNPs into the analysis, we chose additional SNPs across the region until we had similarly captured all SNPs in European Americans with >5% minor allele frequency. All SNPs identified in this way were selected for genotyping on the Sequenom iPLEX platform [43]. Cases and controls were successfully genotyped at a densely spaced panel of 193 tag SNPs across the ∼450 kilobase admixture peak. Each SNP was tested using a χ^2^ statistic assuming an additive effect on neutrophil count for each additional copy of the allele. We did not test a dominant or recessive model because none of the genotyped SNPs (apart from rs2814778) had a frequency differentiation across populations consistent with that SNP explaining the admixture signal (and thus being the main effect SNP; see above). We chose an additive model to search for variants that might modulate the neutrophil count beyond the main effect because it is known that for substantial minor allele frequencies this provides reasonable power to detect an association, whether the true underlying effect is dominant, additive, or recessive [44].

### Analysis of Shotgun Sequencing Data to Rule out the Great Majority of Nucleotides in the Admixture Peak as Containing the Causal Allele

While we found that rs2814778 was more predictive of neutrophil count than ancestry, we were concerned that the variant might not itself be causal, but instead only in LD with the causal variant. To search for additional candidate SNPs in the region that are highly different in frequency between Africans and Europeans, we examined shotgun genome sequence data derived from public databases, from 6 individuals who were known to have West African ancestry across the region (NA18507, NA18517, NA19129, NA19240, NA17109 and NA17119), and 5 individuals who were known to have European ancestry across the region (NA12156, NA12878, NA07340, HuAA and HuBB). These sequences include 4 West Africans and 2 Europeans examined as part of a fosmid end-sequencing project [45], 2 European Americans sequenced as part of the Celera Genomics human genome sequencing project [46],[47], 1 European American sequenced for the purpose of SNP discovery [48],[49], and 2 African Americans also sequenced for SNP discovery [48],[49], who we determined had entirely African ancestry at the locus by using the ANCESTRYMAP software [11] and by unpublished methods (Simon Myers, Alkes Price and Alon Keinan, personal communication). For each individual, we only analyzed nucleotides for which we had a high quality base call at the locus (Neighborhood Quality Score of ≥40) [13]. At sites where we had more than 1× coverage, we randomly sampled one sequence to represent the individual.

To determine the ancestry of the human genome reference sequence across the admixture peak, we first observed that it was spanned by a mosaic of 5 fully sequenced Bacterial Artificial Chromosomes (BACs), each representing a clone of 86,000–196,000 base pairs (http://genome.ucsc.edu). The problem of determining ancestry of the human reference sequence across the region thus amounts to determining the ancestry of each of the clones separately. To do this, we obtained the allele of the human genome reference sequence at each of 284 HapMap SNPs across the region that had been genotyped in both West Africans and European Americans. For each window of 8 consecutive SNPs in HapMap, we calculated the likelihood that the human reference sequence was of African or European ancestry. To determine this likelihood empirically, we compared the findings to 120 phased European chromosomes and 120 phased African chromosomes from the HapMap database, counting the number of matches to the human reference sequence in each population over that window. We conservatively added 1 to the counts of the tested haplotype for each population to preclude an estimate of zero probability for either population. Under the assumption that only European and African ancestry were possible, the results showed with confidence that 3 of the clones were of European ancestry and 2 were of African ancestry (Figure 4).
